# The associations between serum vascular endothelial growth factor, tumor necrosis factor and interleukin 4 with the markers of blood–brain barrier breakdown in patients with paraneoplastic neurological syndromes

**DOI:** 10.1007/s00702-018-1950-9

**Published:** 2018-10-29

**Authors:** Slawomir Michalak, Alicja Kalinowska-Lyszczarz, Joanna Rybacka-Mossakowska, Mikolaj Zaborowski, Wojciech Kozubski

**Affiliations:** 1 Department of Neurochemistry and Neuropathology, Poznan University of Medical Sciences, 49, Przybyszewskiego str., 60-355 Poznan, Poland; 2 Department of Neurology, Poznan University of Medical Sciences, Poznan, Poland; 3 Division of Gynecologic Oncology, Department of Gynecology, Obstetrics and Gynecologic Oncology, Poznan University of Medical Sciences, Poznan, Poland

**Keywords:** Paraneoplastic neurological syndrome, Vascular endothelial growth factor (VEGF), Tumor necrosis factor-alpha (TNF-alpha), Interleukin 4 (IL-4), S-100B, Neuron-specific enolase (NSE)

## Abstract

The blood–brain barrier (BBB) disruption is a critical step in paraneoplastic neurological syndrome (PNS) development. Several cytokines have been implicated in BBB breakdown. However, the exact step-by-step mechanism in which PNS develops is unknown, and the relationship between a systemic neoplasm and BBB is multilevel. The aim of the present study was to examine serum markers of BBB breakdown (S100B protein, neuron-specific enolase, NSE) and concentrations of proinflammatory (TNF-alpha, VEGF) and anti-inflammatory/immunosuppressive cytokines (IL-4), and to establish their interrelationship in patients with PNS. We analyzed 84 patients seropositive for onconeural antibodies that originated from a cohort of 250 cases with suspected PNS. Onconeural antibodies were estimated with indirect immunofluorescence and confirmed with Western blotting. Serum S-100B was estimated using electrochemiluminescence immunoassay. NSE, VEGF, TNF-alpha and IL-4 were analyzed with ELISA. We found that S-100B protein and NSE serum concentrations were elevated in PNS patients without diagnosed malignancy, and S-100B additionally in patients with peripheral nervous system manifestation of PNS. Serum VEGF levels showed several abnormalities, including a decrease in anti-Hu positive patients and increase in PNS patients with typical manifestation and/or central nervous system involvement. Increase in TNF-alpha was observed in patients with undetermined antibodies. To conclude, the presence of paraneoplastic neurological syndrome in seropositive patients does not affect serum markers of BBB breakdown, with the exception of the group without clinically demonstrated malignancy and patients with peripheral manifestation of PNS. S-100B and NSE might increase during early phase of PNS. VEGF may be involved in typical PNS pathophysiology.

## Introduction

Paraneoplastic neurological syndromes (PNS) are considered to be immune-mediated pathology resulting from remote effects of systemic malignancy. One of the aspects of PNS development is the disruption of the blood–brain barrier (BBB) that may facilitate autoimmune reaction against previously sequestered central nervous system (CNS) antigens. Although autoimmune cells can enter the CNS in the absence of BBB disruption once they have been activated in the periphery, which has been demonstrated for anti-Hu syndrome (de Jongste et al. 2013), BBB disintegrity may contribute to propagation of the neuroinflammatory cascade. Such phenomenon is known for demyelinating autoimmunity in the CNS, namely for multiple sclerosis.

The relationship between systemic neoplasm and BBB is complex. The integrity of BBB has been intensively studied in primary and metastatic brain tumors. Several observations were made, including those of a compromised tight junctions structure, enlargement of perivascular space, fenestrations in blood vessels, increased number and activity of pinocytic vacuoles and decreased expression of transporters like P-glycoprotein (PgP) in the endothelial cells (Bart et al. 2000; Liebner et al. 2000; Shibata 1989; Regina et al. 2001).

Mechanisms leading to BBB breakdown involve direct and indirect effects of inflammatory mediators. Cytokines were suggested as contributors of endothelial cells lesion and BBB breakdown in primary CNS lymphomas (Molnár et al. 1999). Local BBB lesions may contribute to the pathogenesis of paraneoplastic neurological syndromes. The indirect effects of cancer leading to BBB breakdown are mediated by numerous factors. Matrix metalloproteinases (MMP) and their endogenous inhibitors (tissue inhibitors of MMPs, TIMPs) are targets of cytokines’ effects on BBB in several neuroinflammatory diseases (Rosenberg 2002). Tumor necrosis factor-alpha (TNF-alpha) is a major proinflammatory cytokine that regulates MMP and TIMP expression (Johnatty et al. 1997). It also upregulates the secretion of proMMP9 by T-lymphocytes (Rosenberg 2002) and upregulates the expression of matrix metalloproteinases not only on T-lymphocytes, but also on endothelial cells (Hanemaaijer et al. 1993). It is emphasized in the literature that TNF plays a particular role as a mediator of MMP expression and synthesis (Chandler et al. 1997).

Interleukin-4 (IL-4) is Th2-type cytokine, considered to be predominantly anti-inflammatory and immunosuppressive. Interleukin-4 is suggested as a critical regulator of CNS autoimmune inflammation (Gomez et al. 1997). It downregulates the secretion of numerous factors including TNF-alpha (Tan et al. 2003). On the other hand, IL-4 was found to stimulate T-cell-induced MMP-1 and inhibit MMP-9 production in monocytes’ cultures (Mannello and Gazzanelli 2001). This cytokine also acts as an inhibitor of matrix metalloproteinase expression induced by interleukin-1 beta (Ponomarev et al. 2007) and TNF-alpha (Hart et al. 1989). The inhibition of prostaglandin E2 (PGE2) synthesis by IL-4 is linked to its inhibition of matrix metalloproteinases’ production (Stewart et al. 2007). A suggestion was provided by an in vivo study (Beppu et al. 2002), that IL-4 may also regulate CNS inflammation by inducing apoptosis of activated microglia and enhancing neuronal survival. Anti-inflammatory activity of IL-4 is also linked to its effects on angiogenesis. Anti-angiogenesis action of IL-4 may result from the effect of fibroblast growth factor receptor-1 (FGFR-1) (Gratchev et al. 2005). However, it also stimulates the expression of vascular endothelial growth factor (VEGF) in endothelial cells (Corcoran et al. 1992). IL-4 inhibits in vitro the chemotactic effects of VEGF on human umbilical vein endothelial cells (HUVEC) (Park et al. 2005).

VEGF is a proangiogenic cytokine, which enhances vascular permeability and increases BBB leakage. It is a key factor responsible for tumor neovascularization (Guyot et al. 2017), with cancer cells overexpressing VEGF, which leads to increased angiogenesis (Senger et al. 1993; Folkman and Angiogenesis 2006). In neurological disorders, VEGF signaling is implicated in stroke, hydrocephalus, but also neurodegenerative disorders, such as Alzheimer’s disease (Shim and Madsen 2018). For autoimmune CNS diseases, the cytokine effect of VEGF expression is proposed. Mechanisms of VEGF effects on BBB breakdown remain uncertain. It was shown in vitro that VEGF downregulates endothelial tight junction proteins, namely claudin-5 and occludin (Argaw et al. 2009). Participation of nitric oxide (NO) is also attributed to this effect (Huang et al. 2007; Mayhan 1999).

To test the connection between serum concentrations of TNF, IL-4 and VEGF and BBB breakdown, we have included in the analysis serum levels of S-100B protein and neuron-specific enolase (NSE), which are established markers of BBB breakdown.

S-100B protein is an acidic Ca^2+^-binding protein with two subtypes: S100A1B form present in astroglial cells, and the S100BB form predominantly found in astroglial cells and Schwann cells (Jönsson et al. 2000; Zimmer et al. 1995). Both these subtypes are brain-specific and their plasma concentrations in physiology are very low and account for about one-third of cerebrospinal fluid levels (Grocott et al. 2001). Therefore, its peripheral elevation is a result of BBB damage. Osmotic disruption of BBB without damage of neuronal cells causes increase of serum S-100B concentration (Kapural et al. 2002).

Neuron-specific enolase (NSE) is referred to as γγ-homodimer or αγ-heterodimer (in neurons) and αα- or ββ-homodimer isoenzymes (in non-neuronal cells) of a glycolytic enzyme–enolase (2-phospho-d glycerate hydrolyase or phosphopyruvate hydratase) (Marangos et al. 1979; Marangos and Schmechel 1987). NSE is considered as a marker of parenchymal brain injury (Marangos et al. 1979). Serum concentrations of NSE in physiological conditions are negligible, therefore, the release into the circulation must occur due to disruption of the BBB. The observation that NSE levels remain unchanged with BBB opening by osmotic means (Marangos and Schmechel 1987) might suggest that both conditions, namely neuronal damage and BBB breakdown, are required for eventual increase of serum NSE concentration.

The purpose of our study was to examine serum markers of BBB breakdown and serum concentrations of proinflammatory (TNF-alpha, VEGF) and anti-inflammatory/immunosuppressive (IL-4) cytokines in patients with paraneoplastic neurological syndromes.

## Materials and methods

Eighty-four patients seropositive for onconeural antibodies originated from a cohort of 250 cases with a clinical suspicion of paraneoplastic neurological syndrome, who were hospitalized or consulted in Departments of Neurology in Poznan, Poland. The study protocol was approved by the Internal Review Board at the Poznan University of Medical Sciences. Written informed consent was obtained from all the study participants. Onconeural antibodies were identified by means of indirect immunofluorescence (EUROIMMUN, Kiel, Germany) and subsequent line blot was performed in positive cases (EUROIMMUN, Kiel, Germany). Only seropositive patients were included in the study. The final diagnosis of paraneoplastic neurological syndromes was based on Graus criteria (Graus et al. 2004). Both definite and possible PNS according to Graus criteria were included in the study. The female/male ratio in the study group was 1.3, and the mean age of the patients was 52 ± 15 years (mean ± SD). Patients were categorized into four groups: with well-characterized onconeural antibodies (anti-Hu, anti-Yo, anti-Ri) and those with unidentified onconeural antibodies as mentioned in Graus’ paper (Graus et al. 2004). Unidentified antibodies showed a positive reaction on Western blotting, however, the bands could not be assigned to anti-Hu, anti-Yo or anti-Ri. We also compared groups of patients with central (subacute cerebellar degeneration, limbic encephalitis, brainstem encephalitis, myelopathy, motor neuron disease) and peripheral manifestations (subacute sensory neuronopathy, acute sensorimotor neuropathy, autonomic neuropathy, neuromyotonia, Lambert–Eaton myasthenic syndrome and myasthenia) of PNS. Classical and non-classical PNS according to Graus (Graus et al. 2004) were taken into consideration, as well. Finally, the patients with a known diagnosis of primary malignancy were compared to those without known neoplasms. All patients were treatment-naïve with regards to immunotherapy or chemotherapy at the time of inclusion into the study.

Serum S-100B protein was estimated using electrochemiluminescence immunoassay (ROCHE Diagnostics, Vilvoorde, Belgium). NSE, VEGF, IL-4 and TNF-alpha were measured with the use of ELISA method, according to manufacturer’s instructions (BIOMEDA, Foster City, CA for NSE, and Bender Medsystem GmbH, Vienna, Austria for VEGF, IL-4 and TNF-alpha).

Statistical analysis was performed using licensed STATISTICA software version 12 (StatSoft Polska sp. z o.o., Kraków, Poland). First, the distribution of variables was tested with the Shapiro–Wilk test. All variables that had non-Gaussian distribution were expressed as median, and interquartile range and analyzed with the Mann–Whitney test.

## Results

### Clinical data

**Fig. 1:**
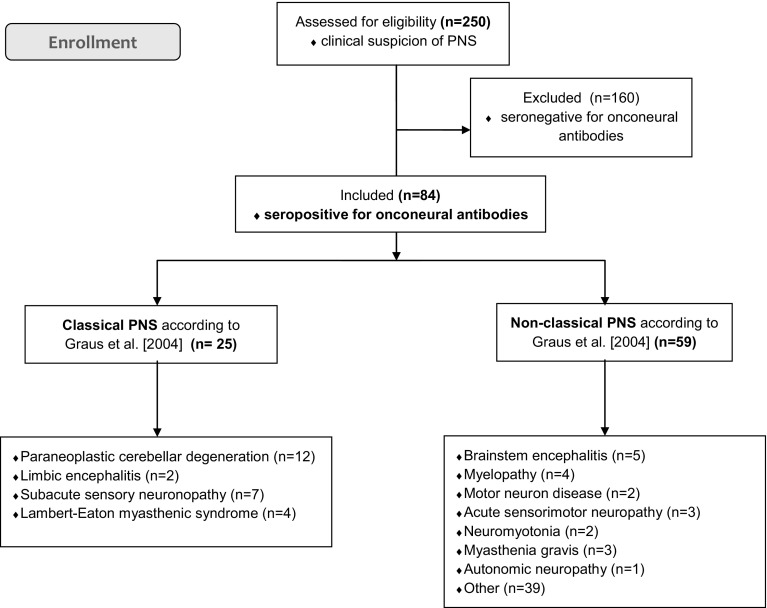
Flow diagram of the study progress. *PNS* paraneoplastic neurological syndrome. According to 2004 Graus criteria classical PNS is defined as: encephalomyelitis, limbic encephalitis, subacute cerebellar degeneration, opsoclonus-myoclonus, subacute sensory neuronopathy, chronic gastrointestinal pseudoobstruction, Lambert–Eaton myasthenic syndrome, dermatomyositis; and non-classical PNS includes: brainstem encephalitis, stiff person syndrome, acute sensorimotor neuropathy (Guillain–Barré syndrome, brachial neuritis), subacute/chronic sensorimotor neuropathies, neuropathy with vasculitis, acute pandysautonomia, acquired neuromyotonia, acute necrotizing myopathy

From a cohort of 250 patients with a clinical suspicion of a paraneoplastic neurological syndrome, 84 patients were seropositive for onconeural antibodies, as indicated in Fig. 1. In this subgroup, we observed both, classical and non-classical clinical manifestations (see Fig. 1), which included: cerebellar degeneration, limbic encephalitis, Lambert–Eaton myasthenic syndrome, neuropathies, myelopathy, brainstem encephalitis, stiff-man syndrome, plexopathy, neuromyotonia and motor neuron disease.

Basing on indirect immunochemistry and subsequent Western blotting performed in positive cases, we found 19 anti-Hu positive subjects (22%), 25 anti-Yo positive (30%), 20 anti-Ri positive (24%) and 20 with unidentified antibodies (24%).

Primary tumors were diagnosed in 23% of seropositive patients and included: breast cancer, ovarian cancer, lung cancer, colorectal cancer, thyroid gland cancer, lymphoma, thymoma and urinary bladder cancer.

Malignancies diagnosed in patients seropositive for well-characterized onconeural antibodies included: breast (8.3%), ovarian (8.3%) and lung cancer (4.2%), non-Hodgkin lymphoma (4.2%), anal (4.2%), endometrial (4.2%), tongue carcinoma (4.2%), adrenal adenoma (4.2%), and paraproteinemia (4.2%).

In patients with unidentified antibodies, the following neoplasms were diagnosed: sigmoid cancer (3.8%), non-Hodgkin lymphoma (3.8%) and lung cancer (3.8%).

Interestingly, we have identified onconeural antibodies in patients with paraneoplastic neurological syndromes who had no malignancy diagnosed yet. In this group, the antibodies profile was as follows: anti-Ri (10.5%), anti-Yo (7.5%), anti-Hu (1.5%) and unidentified (22.5%).

### Neuron-specific enolase and S-100Β levels

We have found that the median level for serum NSE was 0.00 (interquartile range: 0.00–19.87 U/mL) and for S-100 it was 53 (interquartile range: 37–80 µg/mL), which was within reference values (< 25 U/mL, BIOMEDA, Foster City, CA, USA for NSE and < 105 µg/mL, ROCHE Diagnostics, Vilvoorde, Belgium, for S-100). There were no significant differences of NSE and S-100 serum concentrations between groups of patients with anti-Yo, anti-Ri, anti-Hu and unidentified antibodies (see Table 1). NSE level did not differ between patients with central and peripheral manifestation of PNS. On the contrary, S-100 serum concentration was higher (*p* < 0.01) in patients with peripheral manifestation (see Table 1), but still within reference values, which is below 105 µg/mL. We did not find significant differences in NSE and S-100 levels between patients with classical and non-classical PNS, or between patients with well-characterized and unidentified onconeural antibodies (see Table 1). Twenty-five percent of anti-Hu seropositive patients had S-100 levels over reference value. This was true for 20% of anti-Ri, 8% of anti-Yo cases and 5% of patients with unidentified antibodies. No differences in S-100Β protein concentration were found between the subgroups.

**Table 1:**
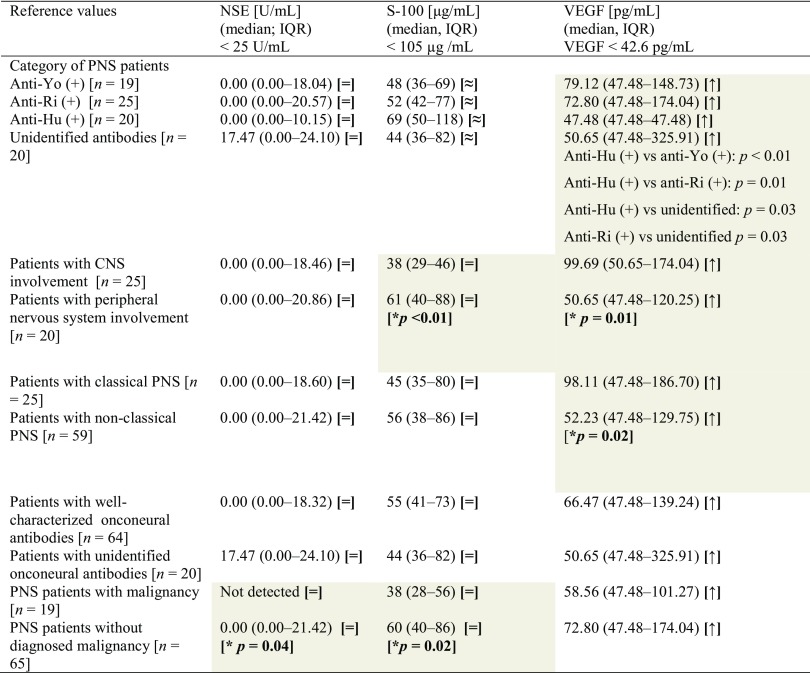
Serum NSE, S-100B and VEGF levels in patients with paraneoplastic neurological syndromes

Statistically significant (*p* ≤ 0.05) differences between subgroups have been marked with gray color and bold type highlighted *p* value

The signs in brackets refer to reference values as follows: [=] within reference values, [≈] almost within reference values (median is within reference, but individual patients exceeded reference values), [↓] below reference values, [↑] above reference values

*PNS* paraneoplastic neurological syndrome, *NSE* neuron-specific enolase, *VEGF* vascular endothelial growth factor, *IQR* interquartile range, *CNS* central nervous system

Interestingly, patients with no malignancy identified had higher levels of both NSE (*p* = 0.04) and S-100Β (*p* = 0.02), although still within reference values.

### Vascular endothelial growth factor (VEGF)

All patients that were seropositive for onconeural antibodies had VEGF above reference values, which are defined as below 42.6 pg/mL (Bender Medsystem GmbH, Vienna, Austria). However, significant differences were observed between the subgroups. Serum concentration of VEGF was the lowest in anti-Hu seropositive patients. Significant differences were found between anti-Ri seropositive patients and patients with unidentified antibodies (Table 1). Patients with central and classical manifestation of PNS presented higher serum VEGF levels than subjects with peripheral and non-classical PNS, respectively (Table 1). Whether the identified antibodies were well-characterized or unidentified, or whether the diagnosis of primary neoplasm was confirmed, did not affect VEGF serum concentration.

### Interleukin-4 (IL-4)

Interleukin-4 serum concentrations did not show differences between the studied subgroups (see Table 2). Reference value for serum IL-4 is defined as absent (Bender Medsystem GmbH, Vienna, Austria).

**Table 2:**
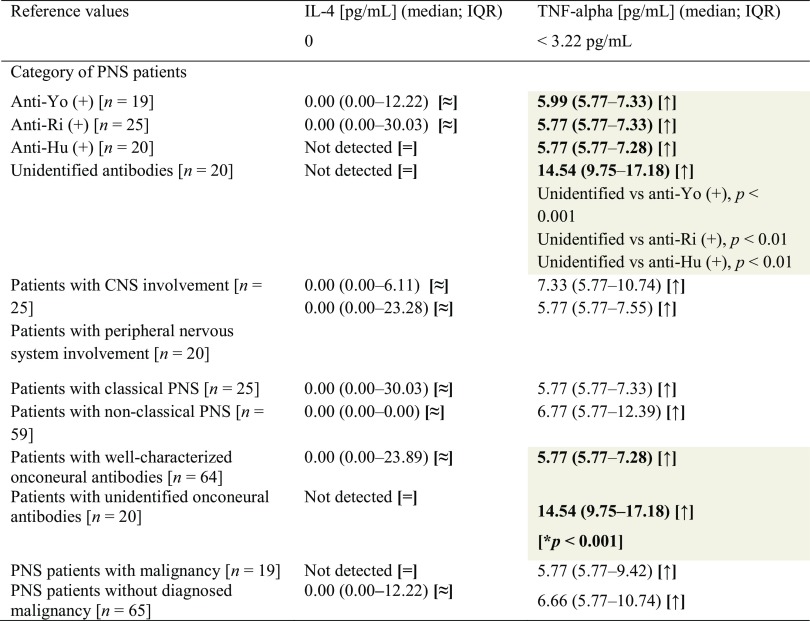
Serum IL-4 and TNF-alpha concentrations in patients with paraneoplastic neurological syndromes

Statistically significant (*p* ≤ 0.05) differences between subgroups have been marked with gray color and bold type highlighted *p* value

The signs in brackets refer to reference values as follows: [=] within reference values, [≈] almost within reference values (median is within reference, but individual patients exceeded reference values), [↓] below reference values, [↑] above reference values

*IL-4* interleukin 4, *TNF-alpha* tumor necrosis factor-alfa, *PNS* paraneoplastic neurological syndromes, *CNS* central nervous system

### Tumor necrosis factor-alpha (TNF-alpha)

All patients that were seropositive for onconeural antibodies had TNF-alpha levels above reference values, which are defined as below 3.22 pg/mL (Bender Medsystem GmbH, Vienna, Austria). However, significant differences were observed between several subgroups. Patients with well-characterized onconeural antibodies had lower serum TNF-alpha concentrations than subjects with unidentified antibodies (see Table 2). We also found higher TNF-alpha levels in anti-Yo positive patients than in those with anti-Ri antibodies.

## Discussion

Blood–brain barrier breakdown remains a critical issue in the immune hypothesis of paraneoplastic neurological syndromes pathogenesis. In our previous studies, we observed BBB breakdown during the course of experimental neoplastic disease (Michalak et al. 2010). However, in clinical studies so far there has been no evidence of BBB disintegrity.

In the present study, we observed that VEGF and TNF-alpha levels in our PNS cohort were higher than those referenced as normative values. As for, S-100B and NSE, on a group level the values did not differ from the healthy reference, however, in individual patients the levels exceeded reference data. Also, there were significant differences between subgroups of PNS patients, suggesting that in some of them BBB breakdown may be of more importance.

In our study, we have shown that serum NSE and S-100Β levels were higher in patients with paraneoplastic neurological syndromes and onconeural antibodies, but without diagnosed malignancy, than in those with localized primary tumors. This may indicate that BBB disruption occurs in the very early stage of neoplastic disease, when it is required for the autoimmune reaction against onconeural antigens. It should be asked what the primary cause of serum NSE and S-100Β elevation is. One may expect it during the very early stage of peripheral tumor growth, and this mechanism may be associated with BBB breakdown. At the PNS stage when systemic cancer is already clinically manifested and onconeural antibodies are present, serum levels of BBB damage markers may no longer be increased.

Also, we have noticed higher S-100Β protein concentrations in patients with peripheral manifestations of PNS, when compared to those with the CNS involvement. S-100Β protein is known to be expressed in Schwann cells in the developing peripheral nerve (Shearman and Franks 1987). It was primarily recognized as specific for the nervous system, however, further studies showed its expression in peripheral blood T4 (Ferrari et al. 1988) and T8 lymphocytes (Takahashi et al. 1985). Should the concept of the role played by T-lymphocytes in PNS pathogenesis be accepted, then serum S100Β concentration could be considered not only as BBB leakage indicator, but also as a marker of immune system activation, which may be the case in our study. A marked decrease in the percentage of S100Β positive lymphocytes was observed in the course of advanced peripheral malignancy and no changes in the early stages of gastric cancer were found when compared with the healthy subjects (Takahashi et al. 1987). Dendritic cells infiltrating colorectal tumors express S-100Β protein, as well (Sandel et al. 2005). The clinical significance of S-100Β positive dendritic cells infiltration is equivocal. Ambe et al. showed a positive correlation between the number of infiltrating dendritic cells and a favorable clinical outcome of colorectal cancer (Ambe et al. 1989). However, this was not the case in other studies in colorectal cancer (Dadabayev et al. 2004) and also in breast, gastric, ovarian, and tongue carcinomas (Goldman et al. 1998; Lespagnard et al. 1999; Okuyama et al. 1998; Eisenthal et al. 2001; Iwamoto et al. 2003).In melanoma patients, increased serum S-100Β levels (Schultz et al. 1998) correlated with clinical stage, prognosis, and survival. The expression of S-100Β protein has also been found in T-cell lymphoproliferative disorder (Zarate-Osorno et al. 1994).

We did not observe serum NSE increase in our PNS cohort. Moreover, the levels were higher in patients without known malignancy than in patients with tumor diagnosis established. In the literature, NSE levels were found to be elevated in 78% of patients with small-cell lung cancer (SCLC) (Burghuber et al. 1990). Elevation of serum NSE levels was also observed for tumors originating from neuroendocrine cells (APUD) and neuroblastoma (D’Alessandro et al. 1992; Zeltzer et al. 1986). In our study, we hypothesized that proinflammatory (TNF-alpha), anti-inflammatory (IL-4) and proangiogenic (VEGF) cytokines may play a role during development of PNS and may affect BBB integrity.

Indeed, we have found VEGF to be increased in all of our PNS patients, however, significantly more in PNS patients with CNS manifestations, as well as with the classical PNS. This may suggest involvement of VEGF in the pathogenesis of a particular subgroup of PNS clinical manifestations. VEGF exerts effects, which may be involved in tumor-associated autoimmunity. When expressed on tumor cells, VEGF blocks dendritic cells differentiation and maturation, leading to accumulation of immature dendritic and immature myeloid cells (Gabrilovich et al. 1996; Midgley and Kerr 2005). To our knowledge, this is the first study examining serum VEGF in PNS patients. However, VEGF was identified as a factor produced by a number of systemic malignancies, including lung cancer, ovarian and breast cancer (Kraft et al. 1999; Ohta et al. 1996; Olson et al. 1994; Brown et al. 1995). In our study, we were not able to find differences in serum VEGF concentrations between patients with and without clinically manifested malignancy. Therefore, higher VEGF levels in patients with central and classical syndromes rather indicate its involvement in the pathogenesis of PNS, and not with the primary tumor itself. Noteworthy, VEGF concentration was lower in patients seropositive for anti-Hu antibodies, when compared with patients with other onconeural antibodies. Experimental studies showed that VEGF decreases T-cells number and T-cell-to-B-cell ratio in lymph nodes and spleen of non-tumor-bearing mice (Gabrilovich et al. 1998). VEGF induces thymic atrophy, decreases number of thymocytes and impairs T-cell development from early hematopoietic progenitor cells (Ohm et al. 2003). On the other hand, VEGF upregulates proinflammatory T-cells differentiation, as evidenced in EAE, which has earlier onset, more severe and prolonged manifestation when induced by VEGF-stimulated T-cells comparing to intact T-cells (Mor et al. 2004). Therefore, VEGF directs T-cells into Th1 proinflammatory mode and further enhances inflammatory reaction. VEGF does not affect motility of normal B-lymphocytes (Till et al. 2005), however, its effect on other functions remains obscure. Paraneoplastic neurological syndromes with anti-Hu antibodies are associated with stimulation of cytotoxic T-lymphocytes (Voltz et al. 1998). Helper and cytotoxic T-lymphocytes reacting with Hu antigen were found in peripheral blood of PNS patients (Benyahia et al. 1999; Tanaka et al. 1999). In our study, decreased serum VEGF concentration in anti-Hu positive PNS patients may indicate disturbed regulation of the above-mentioned mechanisms. Other studies (Bouzin et al. 2007) revealed the inhibitory effect of VEGF on lymphocytic adhesion on activated endothelial cells. Decreased serum VEGF concentration observed in our group of anti-Hu and classical PNS patients may participate in adhesion and penetration of activated lymphocytes through BBB.

VEGF, together with interleukin-10, TNF-alpha and TGF-beta, belongs to cytokines responsible for tumor-associated immunodeficiency (Ohm and Carbone 2001). TNF-alpha acts synergically with VEGF, as it was demonstrated in case of the tissue factor production by endothelial cells (Mechtcheriakova et al. 2001). Moreover, TNF stimulates VEGF production by human progenitor cells from bone marrow (Menetrier-Caux Ch, Thomachot et al. 2001). Co-culture of murine bone marrow cells with tumor culture conditioning medium caused increased production of both, VEGF and TNF (Canque et al. 1998). TNF, as opposed to VEGF, increases lymphocytic adhesion to endothelial cells (Bouzin et al. 2007). We were not able to show any correlations between serum VEGF and TNF concentrations in our cohort. Also, in our study, serum concentration of TNF was higher in patients with unidentified antibodies. Therefore, in this subgroup of patients one may expect enhanced adhesion of lymphocytes to endothelium and further passage across BBB. However, adequate interpretation of this finding needs further detailed studies, particularly identification of remaining onconeural antibodies, which is one of the future directions that could shed more light on our results. It would be interesting to include other markers of BBB damage, namely transmembrane tight junction proteins, such as occludin or claudin-5.

Interleukin-4 was the only factor examined in our study, which did not show any differences between the studied groups of patients. We included IL-4 in the analysis, because of its anti-inflammatory and immunosuppressive effects, which showed promise with regards to remote immunological effects of systemic malignancy. However, in our cohort we were not able to confirm IL-4 involvement in PNS pathomechanism. This could be explained by the fact that all patients were examined at the seropositive stage of PNS development, and the regulatory role of IL-4 appears in the very early, preclinical phase.

One of the limitations that we need to recognize is that we did not have cerebrospinal fluid (CSF) data in all subjects in our cohort. Therefore, we could not calculate CSF/serum albumin ratio, which is a standard way to demonstrate BBB disruption, which could add context to our data. Also, since reference values are known for the markers we examined, we did not include a control group. However, adding another type of a control group, such as patients with other non-inflammatory neurologic disorders, or patients with known malignancy but without paraneoplastic syndrome, would definitely broaden the perspective of our study.

To test the relevance of our data, it would be interesting to assess how the above-mentioned markers responded to cancer therapy, and correlate it with clinical data, including neurological and general prognosis.

To conclude, paraneoplastic neurological syndromes in patients seropositive for onconeural antibodies are not associated with significant alterations of serum NSE and S-100B levels, with the exception of the subgroup without clinically active malignancy and also, for S-100Β only, in the subgroup of patients with peripheral PNS manifestation. Therefore, we suggest that both markers of blood–brain barrier disruption might increase during the early phase of PNS. The changes of serum VEGF concentrations, that we observed in this study, indicate its association with the pathomechanism of PNS with a typical manifestation, and also with the involvement of CNS and production of anti-Hu antibodies. In the view of extensive studies on the effects of VEGF antagonists in cancer patients, the latter observation may open new treatment possibilities.

## Abbreviations

BBB: Blood–brain barrier
CNS: Central nervous system
EAE: Experimental autoimmune encephalitis
FGFR-1: Fibroblast growth factor receptor-1
HUVEC: Human umbilical vein endothelial cells
IFN-gamma: Interferon-gamma
IL-1 beta: Interleukin-1 beta
IL-4: Interleukin 4
IL-6: Interleukin 6
MIP: Macrophage inflammatory protein
MMP: Matrix metalloproteinases
NO: Nitric oxide
NSE: Neuron-specific enolase
PGE2: Prostaglandin E2
PgP: P-glycoprotein
PNS: Paraneoplastic neurological syndrome
RANTES: Regulated on activation normal T-cell expressed and secreted
SCLC: Small-cell lung cancer
TIMPs: Tissue inhibitors of matrix metalloproteinases
TNF-alpha: Tumor necrosis factor-alpha
VEGF: Vascular endothelial growth factor
